# The Polyphenol-Enriched *Salix songarica* Extract Alleviate High-Fat, High-Cholesterol Diet-Induced Inflammation via the LPC18:1/GPR120/β-Arrestin-2/NLRP3 Signaling Pathway

**DOI:** 10.3390/foods15173010

**Published:** 2026-08-26

**Authors:** Hao Li, Nuoyu Ma, Xuemei Dai, Ruixin Lin, Zhuo Yao, Xiaoqi Yan, Shangzhi Xu, Yunhua Hu, Wenwen Zhang, Haixia Wang, Qingzhe Yang, Xinyong Guo, Li Zhang

**Affiliations:** 1 Department of Preventive Medicine, School of Public Health, Shihezi University, Shihezi 832000, China; 2 College of Life Science, Shihezi University, Shihezi 832000, China; 3 Key Laboratory for Prevention and Control of Emerging Infectious Diseases and Public Health Security, The Xinjiang Production and Construction Corps, Urumqi 830002, China

**Keywords:** *Salix songaraca* Andersson, polyphenol-enriched extract, inflammatory response, LPC, GPR120

## Abstract

*Salix songarica* Andersson (*S. songarica*), a medicinal plant endemic to Xinjiang, exhibits high polyphenol content in its floral tissues. Although it shows anti-inflammatory potential, the underlying mechanisms remain unclear. This study investigated the therapeutic effects of polyphenol-enriched *S. songarica* flower extract (SSFP) on inflammation and dyslipidemia in high-fat, high-cholesterol diet (HFHCD)-fed ApoE^−/−^ mice. SSFP administration significantly improved serum lipid profiles, hepatic function, and systemic inflammatory biomarkers in these mice, while also attenuating histopathological inflammation in the liver, kidney, and colon. Furthermore, SSFP treatment upregulated the expression of GPR120 and β-arrestin-2 and suppressed NLRP3 inflammasome activation. In vitro experiments further showed that the anti-inflammatory effect of LPC18:1 was consistent with SSFP. Together, our results demonstrate that SSFP alleviates inflammation through the LPC18:1/GPR120/β-arrestin-2/NLRP3 signaling axis, supporting its potential application as a natural functional food ingredient.

## 1. Introduction

Inflammation constitutes a fundamental physiological defense mechanism against pathogenic invasion and tissue injury [1,2]. However, its persistent or dysregulated activation promotes chronic low-grade inflammation, a key pathological feature underlying inflammatory bowel disease, metabolic syndrome, and cardiovascular disorders [3]. In recent years, natural plant-derived polyphenols have garnered considerable scientific and translational interest as functional food constituents, owing to their well-documented anti-inflammatory properties and capacity to modulate metabolic homeostasis [4,5,6]. *Salix songarica* Andersson (*S. songarica*) is a Xinjiang-endemic plant species with well-established dual attributes of medicinal utility and dietary applicability. The *S. songarica* flower is a chrysanthemum-like morphological structure arising from aberrant proliferation of young branches and floral buds, typically induced by viral infection or insect herbivory, and is notably enriched in bioactive polyphenols including epicatechin, luteolin, and gallic acid. Traditionally employed in herbal tea preparations and health-promoting formulations, *S. songarica* holds considerable promise for integration into the nutritional and functional food sector [7,8,9,10]. Nevertheless, current scientific inquiry into the anti-inflammatory properties of polyphenol-enriched *S. songarica* flower extract (SSFP) remains limited, and the underlying molecular mechanisms have yet to be systematically elucidated.

The NLRP3 inflammasome serves as a central signaling hub within the innate immune system, and its activation triggers the proteolytic maturation and secretion of the proinflammatory cytokines murine interleukin-1β (IL-1β) and murine interleukin-18 (IL-18) [11,12]. Dysregulated overactivation of the NLRP3 inflammasome is strongly implicated in the pathogenesis of chronic inflammatory conditions and represents a therapeutically relevant target for anti-inflammatory intervention [13]. As a G protein-coupled receptor, GPR120 orchestrates both metabolic homeostasis and anti-inflammatory responses. On one hand, it modulates lipid and glucose homeostasis [14,15,16,17,18]. On the other hand, upon ligand binding, it recruits β-arrestin-2 to suppress canonical proinflammatory signaling, including NF-κB activation, while concurrently inhibiting the assembly of the NLRP3 inflammasome, maturation of caspase-1, and subsequent secretion of IL-1β [19,20,21,22]. These findings collectively highlight the GPR120/NLRP3 axis as a mechanistically plausible anti-inflammatory target. A key unanswered question, however, is whether exogenous compounds can be developed to selectively modulate this pathway.

Lysophosphatidylcholine (LPC), a bioactive lipid metabolite derived from phosphatidylcholine hydrolysis [23], exhibits subtype-specific immunomodulatory effects, with distinct anti-inflammatory or pro-inflammatory activities attributable to structural variations among its molecular species [24]. Specific molecular species, including LPC 14:0 and LPC 18:0, have been shown to inhibit NLRP3 inflammasome activation and exert anti-inflammatory effects [25,26,27,28], suggesting their role as potential endogenous ligands for GPR120 and their upstream activators of downstream signaling cascades. Our previous independent experiments using UPLC-MS/MS-based lipidomics analysis revealed that intervention with *S. songarica* flower extract upregulated the levels of multiple LPC subtypes in the serum of obese mice, suggesting that LPC may be involved in the anti-inflammatory effects of *S. songarica* flower. However, the key LPC subtypes regulated by SSFP and their underlying molecular mechanisms remain unclear.

Although exogenous LPC has been proven to possess anti-inflammatory potential [25,26,27,28], it remains unclear whether natural polyphenols exert anti-inflammatory effects by reshaping the endogenous LPC metabolic profile. Furthermore, although polyphenol monomers such as epicatechin and hedyotis diffusa glycosides contained in SSFP can synergistically intervene in inflammatory signaling [29,30,31], their overall effects are still unclear. Therefore, this study utilizes the HFHCD-fed ApoE^−/−^ mouse model and the LPS-induced RAW264.7 cell inflammation model to systematically evaluate the anti-inflammatory effects of SSFP and its regulatory mechanism on the GPR120/β-arrestin-2 pathway, screen key anti-inflammatory LPC subtypes, and verify their functional roles in the GPR120 signaling pathway. This study is the first to verify the anti-inflammatory effects of SSFP and its association with the LPC18:1/GPR120/β-arrestin-2/NLRP3 pathway at the whole level, providing a theoretical basis for subsequent mechanistic analysis of active monomers, functional food development of *S. songarica* flower, and dietary intervention for chronic inflammation, while also offering scientific references for the high-value utilization of local characteristic plant resources.

## 2. Materials and Methods

### 2.1. Samples and Reagents

Plant material: Flowers of *Salix songarica* Andersson (*S. songarica*) were harvested from Gurtu Town, Wusu City, Xinjiang Uygur Autonomous Region, People’s Republic of China (44°26′ N, 83°15′ E). The voucher specimen (accession No. 5341) has been deposited in the Herbarium of Shihezi University (SHI). Extraction and purification: Polyphenolic extracts from *S. songarica* flowers were prepared via ultrasound-assisted extraction using 40% aqueous ethanol (50 °C, 40 min, solid-to-liquid ratio of 1:10 g/mL), followed by purification on macroporous adsorption resin and lyophilization to yield a stable, free-flowing freeze-dried powder. Sample preparation for biological assays: Prior to in vivo administration, the freeze-dried polyphenolic extract was reconstituted in sterile 0.9% saline solution. For in vitro cellular studies, it was dissolved in dimethyl sulfoxide at a final concentration not exceeding 0.1% to ensure solvent compatibility with cell viability.

Reagents and dietary materials: A HFHCD, formulated to deliver 60% of total caloric energy from fat and containing 2% cholesterol, alongside a standard rodent chow diet, were procured from Beijing Huafukang Biotechnology Co., Ltd. (Beijing, China). Biochemical assay kits: Enzyme-linked immunosorbent assay (ELISA) kits for serum lipopolysaccharide (LPS) quantification and colorimetric assay kits for total cholesterol (TC), triglycerides (TG), alanine aminotransferase (ALT), aspartate aminotransferase (AST), and Free Fatty Acid (FFA) were acquired from Nanjing Jiancheng Bioengineering Institute (Nanjing, China). Immunoassay and molecular biology reagents: ELISA kits for quantification of IL-1β, IL-18, interleukin-6 (IL-6), tumor necrosis factor-α (TNF-α), and monocyte chemoattractant protein-1 (MCP-1) were procured from Shanghai Jianglai Biotechnology Co., Ltd. (Shanghai, China). Radioimmunoprecipitation assay (RIPA) lysis buffer, bicinchoninic acid (BCA) protein assay kit, and sodium dodecyl sulfate–polyacrylamide gel electrophoresis (SDS-PAGE) gel preparation reagents were obtained from Beijing Solarbio Science & Technology Co., Ltd. (Beijing, China). Primary antibodies—polyclonal anti-mouse GPR120, β-arrestin-2, NLRP3, caspase-1, phosphorylated extracellular signal-regulated kinase 1/2 (p-ERK1/2), total ERK1/2 (t-ERK1/2), IL-6, and IL-1β—and HRP-conjugated goat anti-mouse IgG secondary antibody were sourced from Wuhan SanYing Biotechnology Co., Ltd. (Wuhan, China). All supplementary chemicals were of analytical-grade purity.

Animal model: Ten 8-week-old male C57BL/6J wild-type mice and forty 8-week-old ApoE^−/−^ mice were utilized. Both strains were procured from Beijing Sibeifu Biotechnology Co., Ltd. (Beijing, China). All animals were maintained under SPF conditions (22 ± 2 °C, 50–60% relative humidity, 12 h light/dark cycle) with ad libitum access to standard chow diet and sterile drinking water. All experimental procedures involving animals were conducted in strict accordance with the National Institutes of Health Guide for the Care and Use of Laboratory Animals and were formally approved by the Institutional Animal Care and Use Committee of the First Affiliated Hospital, School of Medicine, Shihezi University (Approval No.: A2025-239).

Cell culture: RAW264.7 murine macrophages, which were authenticated by short tandem repeat profiling and sourced from Wuhan Primecell Life Technology Co., Ltd. (Wuhan, China), were maintained in Dulbecco’s Modified Eagle Medium supplemented with 15% heat-inactivated fetal bovine serum and 1% penicillin-streptomycin solution under humidified atmospheric conditions at 37 °C and 5% CO_2_.

### 2.2. Experimental Design and Intervention Regimen

Following a 7-day acclimatization period, C57BL/6J wild-type mice were assigned to the normal diet control group (ND) and maintained on a standard rodent chow diet. ApoE^−/−^ mice were maintained on a HFHCD and subsequently randomized into four experimental cohorts: the HFHCD-only control group, the low-dose SSFP preventive group (L), the high-dose SSFP preventive group (H), and the SSFP therapeutic intervention group (T). Based on previous research and literature review [32,33,34,35], mice assigned to the L, H, and T groups received daily oral gavage administration of SSFP at dosages of 50, 100, and 200 mg/kg body weight, respectively. The T group commenced SSFP administration via oral gavage following an 8-week HFHCD feeding period. The ND and HFHCD groups received daily oral gavage of sterile 0.9% sodium chloride solution (10 mL/kg body weight) as vehicle controls. All mice were maintained on their respective dietary regimens for a total duration of 16 weeks.

### 2.3. Biological Sample Collection and Processing

All mice underwent a 12 h overnight fast prior to euthanasia. Blood was collected via cardiac puncture under intraperitoneal anesthesia with sodium pentobarbital (30 mg/kg, 0.3% solution), and serum was isolated by centrifugation at 3000× *g* for 15 min at 4 °C for subsequent biochemical and inflammatory biomarker analyses. Liver, kidney, and colon tissues were aseptically excised, trimmed of adherent adipose tissue, and individually weighed. The relative organ weight index was calculated as: organ index (%) = (organ wet weight/terminal body weight) × 100. Tissues designated for histopathological analysis were fixed in 4% paraformaldehyde solution, while the remaining specimens were snap-frozen in liquid nitrogen and stored at −80 °C until further analysis.

### 2.4. Quantification of Serum Biochemical Parameters, Systemic Inflammatory Biomarkers, and Hepatic Functional Indices

Serum concentrations of TC, TG, LPS, FC, and CE were quantified using commercially available assay kits (Nanjing Jiancheng Bioengineering Institute and Beyotime Biotechnology, Nanjing, China), in accordance with the manufacturers’ instructions. Hepatic injury biomarkers, including ALT, AST, and FFA, were similarly measured in serum samples. Serum concentrations of the pro-inflammatory cytokines IL-1β, IL-6, TNF-α, IL-18, and MCP-1 were quantified using ELISA.

### 2.5. Histopathological Evaluation of Tissue Sections

Liver, colon, and kidney tissues were fixed in 4% paraformaldehyde solution for 48 h at room temperature. Following fixation, tissues underwent graded ethanol dehydration, paraffin embedding, deparaffinization, and hematoxylin and eosin (H&E) staining to enable microscopic assessment of architectural integrity and inflammatory cell infiltration. A subset of liver specimens was embedded in optimal cutting temperature compound and fixed in 10% neutral buffered formalin prior to Oil Red O staining for quantitative assessment of hepatic lipid droplet accumulation. H&E- and Oil Red O-stained sections were examined under a light microscope following coverslipping, and the percentage area of positively stained structures was quantified using ImageJ software. Three animals per experimental group were randomly selected for histomorphometric analysis.

### 2.6. Cell Viability Assessment via CCK-8 Assay

RAW264.7 murine macrophages were seeded in 96-well tissue culture plates and allowed to adhere overnight prior to exposure to serially diluted concentrations of SSFP, LPC species (LPC14:0, LPC16:0, and LPC18:1), AH7614, or TUG-891 for 24 h. Reagents were added to each well in accordance with the manufacturer’s instructions for the CCK-8, and absorbance was measured at 450 nm using a microplate spectrophotometer. Selection of LPC molecular species was guided by existing literature evidence and by the modulatory effects of the *S. songarica* flower extract on endogenous LPC profiles.

### 2.7. In Vitro Cell Grouping and Pharmacological Intervention

RAW264.7 murine macrophages were harvested during the logarithmic growth phase, upon achieving 80–90% confluence and exhibiting robust morphological viability. RAW264.7 murine macrophages were stimulated with LPS at a final concentration of 1 μg/mL for 24 h to establish an in vitro model of inflammatory injury. Experimental groups were designated as follows: (1) untreated control group (cultured under standard conditions); (2) LPS-induced inflammatory injury group; (3) LPC18:1–treated group (10 μM); (4) GPR120 antagonist–pretreated group (1 μM AH7614 followed by 10 μM LPC18:1); (5) GPR120 agonist–treated group (10 μM TUG-891); (6) low-concentration SSFP intervention group (25 µg/mL); and (7) high-concentration SSFP intervention group (50 µg/mL). All pharmacological agents were administered 2 h prior to LPS stimulation, and cultures were maintained for an additional 24 h. Subsequently, both cellular lysates and supernatants were harvested for downstream molecular and biochemical analyses.

### 2.8. Quantification of NO, IL-6, and TNF-α in Cellular Supernatants

Supernatants were harvested from cultured cells in each experimental group. NO concentrations in cell culture supernatants were quantified using the Griess reaction. IL-6 and TNF-α concentrations in cell culture supernatants were quantified using commercially available ELISA kits, in strict accordance with the manufacturers’ protocols.

### 2.9. Immunofluorescence Microscopy of Cultured Cells

RAW264.7 murine macrophages were seeded onto sterile glass coverslips placed in 24-well tissue culture plates and subsequently fixed with 4% paraformaldehyde for 15 min at room temperature. Cells were then permeabilized with 0.1% Triton X-100 for 10 min at room temperature, followed by blocking with 5% bovine serum albumin for 60 min. Primary antibody against NLRP3 (diluted 1:200 in blocking buffer) was applied and incubated overnight at 4 °C. On the following day, Cy3-conjugated secondary antibody (diluted 1:400 in blocking buffer) was applied and incubated for 60 min at room temperature in the dark. Nuclei were counterstained with DAPI for 5 min at room temperature, and coverslips were subsequently mounted onto glass microscope slides using an aqueous mounting medium. Fluorescence images were acquired using a laser scanning confocal microscope and subjected to semi-quantitative analysis with ImageJ software (version 1.54p, NIH, Bethesda, MD, USA).

### 2.10. Western Blot Analysis of Target Protein Expression

Protein extraction: RIPA lysis buffer (diluted 1:10 in weight-to-volume ratio), supplemented with 1 mM PMSF and a commercially available protease inhibitor cocktail, was added to 100 mg of freshly harvested animal liver tissue samples. Tissue homogenization was performed on ice using a mechanical homogenizer, followed by probe sonication at 100 W for 2 min with intermittent pulsing to mitigate thermal denaturation. For RAW264.7 macrophages subjected to experimental treatment, cells were washed twice with ice-cold PBS, lysed on ice for 30 min in RIPA lysis buffer supplemented with a protease inhibitor cocktail, and subsequently subjected to probe sonication at 80 W for 1 min using intermittent pulsing. Both tissue homogenates and cell lysates were centrifuged at 12,000× *g* for 20 min at 4 °C, and the supernatants were carefully collected. Protein concentration was quantified using the BCA assay.

Western blot (WB) analysis: Protein samples were resolved by SDS-polyacrylamide gel electrophoresis (8% resolving gel for NLRP3; 12% resolving gel for all other target proteins) and subsequently electrotransferred onto PVDF membranes using a wet transfer system. Membranes were blocked for 2 h at room temperature with 5% non-fat dry milk or BSA in Tris-buffered saline containing 0.1% Tween-20 (TBST), followed by overnight incubation at 4 °C with the respective primary antibodies—GPR120 (1:1250), β-arrestin-2 (1:27,500), NLRP3 (1:7000), caspase-1 (1:7000), IL-6 (1:1250), IL-1β (1:6000), p-ERK1/2 (1:1000), and t-ERK1/2 (1:5000). Following three washes with Tris-buffered saline containing 0.1% Tween-20 (TBST), membranes were incubated for 2 h at room temperature with the respective HRP-conjugated secondary antibodies. Immunoreactive bands were visualized using a high-sensitivity ECL substrate and digitally acquired using a CCD-based gel documentation system. β-actin (1:60,000) or GAPDH (1:275,000) served as the loading control. Band intensities were quantified using ImageJ software (version 1.54p, NIH, Bethesda, MD, USA), and relative protein expression levels were calculated as the ratio of the integrated optical density of each target protein band to that of the corresponding loading control.

### 2.11. Statistical Analysis

Statistical analysis and graphing were performed using GraphPad Prism software (version 10.2, GraphPad Software, San Diego, CA, USA). Data are expressed as mean ± SEM. Group differences were analyzed using one-way analysis of variance (ANOVA) followed by Tukey’s post hoc test for multiple comparisons. Statistical significance was defined as *p* < 0.05.

## 3. Results

### 3.1. SSFP Ameliorates HFHCD–Induced Dyslipidemia and Systemic Inflammation in Mice

**Figure 1:**
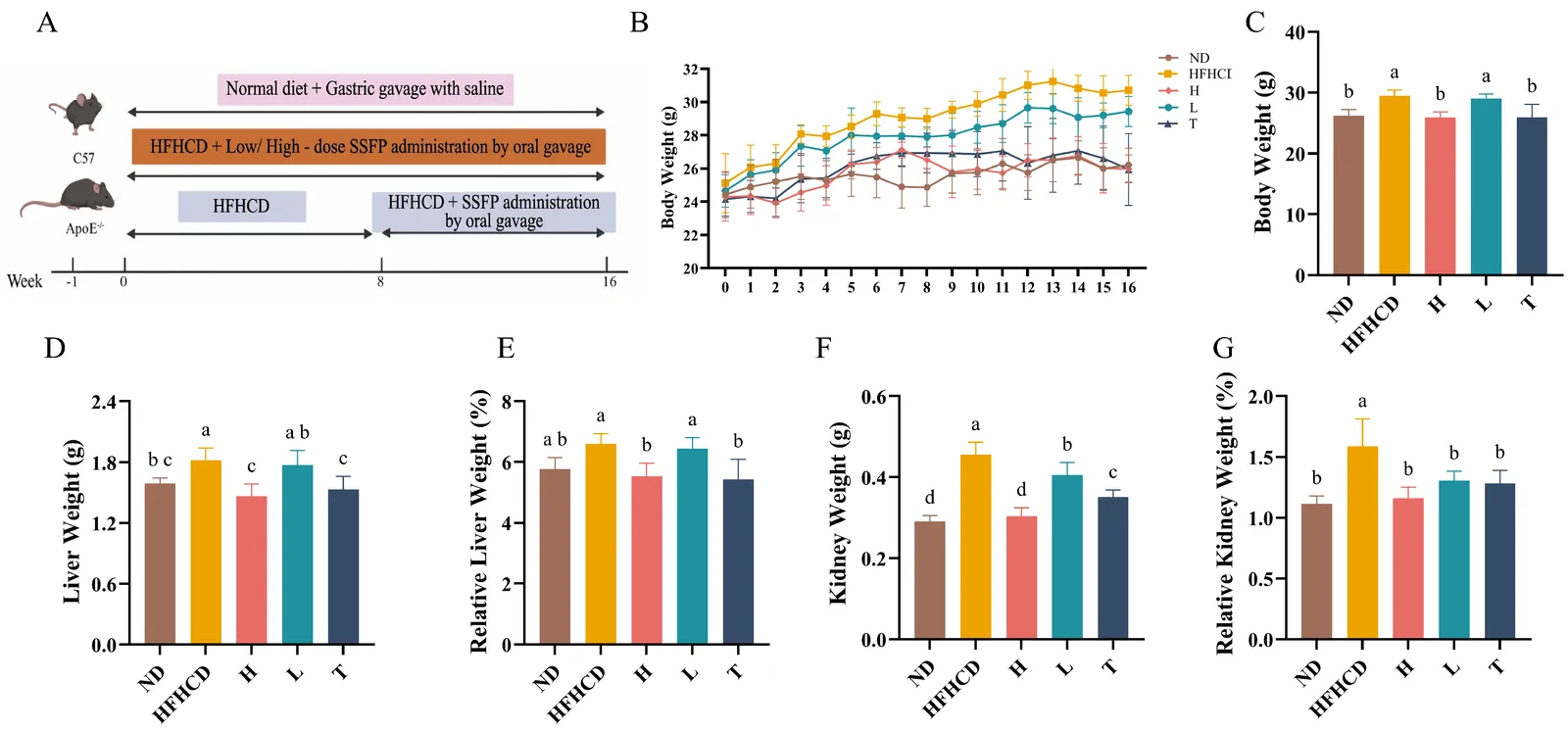
Effects of SSFP on body weight and absolute/relative organ weights in HFHCD-fed ApoE^−/−^ mice. (**A**) Experimental timeline; (**B**) Longitudinal body weight trajectory; (**C**) Final body weight; (**D**) Absolute liver weight; (**E**) Relative liver weight; (**F**) Absolute kidney weight; (**G**) Relative kidney weight. ND: normal diet group; HFHCD: high-fat, high-cholesterol diet model group; L: HFHCD + SSFP low-dose prevention group (50 mg/kg); H: HFHCD + SSFP high-dose prevention group (100 mg/kg); T: HFHCD + SSFP treatment group (200 mg/kg, intervention starting from the 8th week). Statistical significance versus the HFHCD group was determined by one-way ANOVA with Tukey’s post hoc test: data are means ± SE, n = 3. Different lowercase letters indicate significant differences among groups at the same treatment condition (*p* < 0.05).

To assess the ameliorative effects of SSFP on systemic inflammation and dyslipidemia in HFHCD-fed ApoE^−/−^ mice, we first monitored body weight and organ weight indices, and quantified serum lipid profiles and hepatic function biomarkers. Phenotypic analyses revealed that final body weight, as well as absolute and relative weights of the liver and kidneys, were significantly elevated in the HFHCD group relative to the ND group (*p* < 0.05; Figure 1B–G), indicating HFHCD-induced systemic metabolic dysregulation characterized by excessive weight gain and organomegaly. SSFP intervention significantly reduced both absolute and relative liver and kidney weights relative to the HFHCD group (*p* < 0.05; Figure 1D–G), with the most pronounced amelioration observed in the H and T groups.

**Figure 2:**
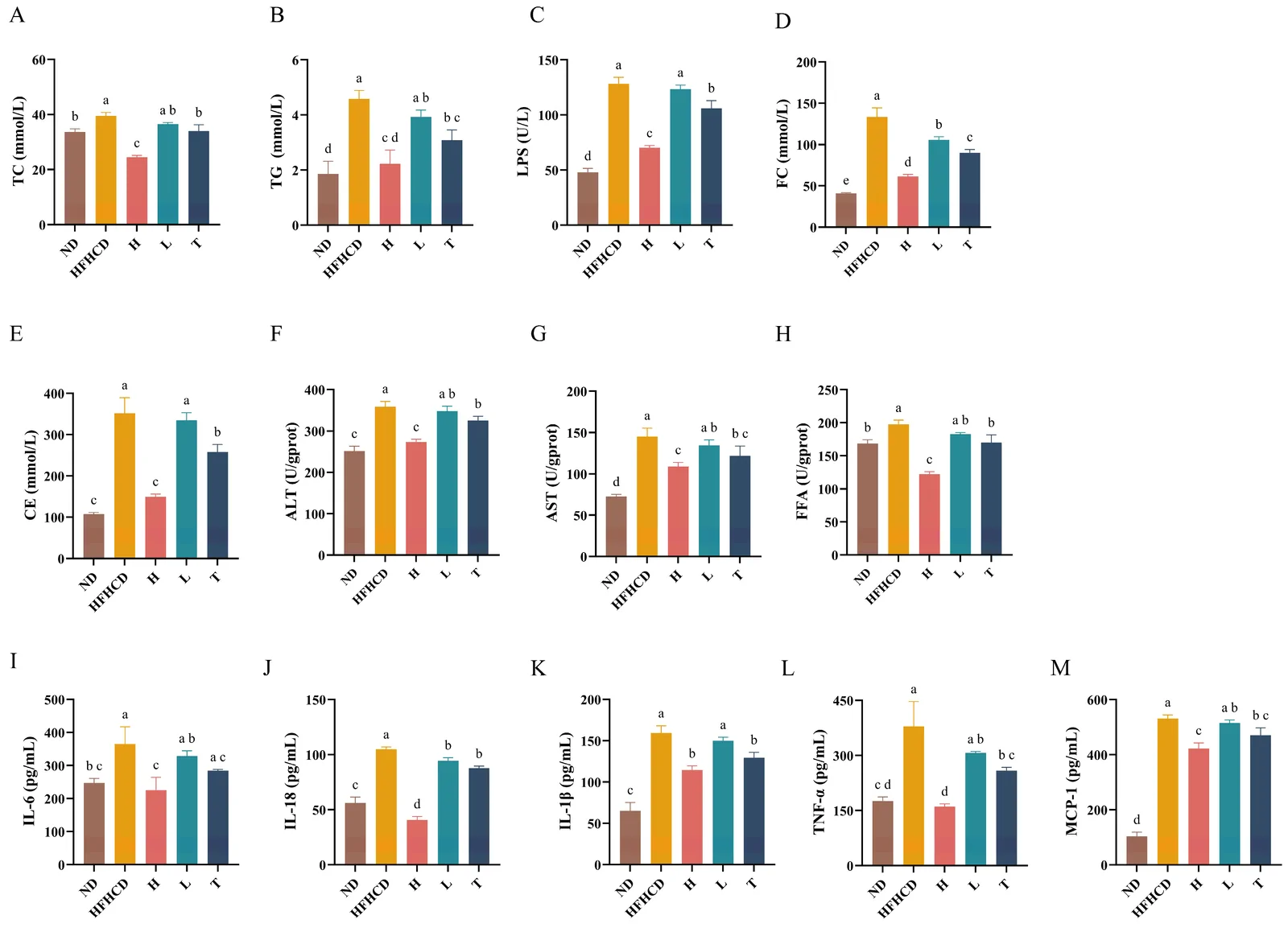
Effects of SSFP on serum and hepatic biochemical parameters and systemic inflammatory mediators in HFHCD-fed ApoE^−/−^ mice. (**A**) Serum TC; (**B**) Serum TG; (**C**) Serum FC; (**D**) Serum CE; (**E**) Serum LPS; (**F**) Hepatic ALT; (**G**) Hepatic AST; (**H**) Hepatic FFA; (**I**) Serum IL-6; (**J**) Serum IL-18; (**K**) Serum IL-1β; (**L**) Serum TNF-α; (**M**) Serum MCP-1. ND: normal diet group; HFHCD: high-fat, high-cholesterol diet model group; L: HFHCD + SSFP low-dose prevention group (50 mg/kg); H: HFHCD + SSFP high-dose prevention group (100 mg/kg); T: HFHCD + SSFP treatment group (200 mg/kg, intervention starting from the 8th week). Statistical significance versus the HFHCD group was determined by one-way ANOVA with Tukey’s post hoc test: data are means ± SE, n = 3. Different lowercase letters indicate significant differences among groups at the same treatment condition (*p* < 0.05).

Biochemical analyses of serum and hepatic lipid parameters further corroborated these observations. Serum concentrations of TC, TG, FC, CE, and LPS were significantly elevated in the HFHCD group relative to the ND group (*p* < 0.05; Figure 2A–E), indicating HFHCD-induced dyslipidemia and metabolic endotoxemia. Moreover, serum biomarkers of hepatocellular injury, including ALT, AST, and FFA, were significantly elevated in the HFHCD group relative to the ND group (*p* < 0.05; Figure 2F–H), indicating HFHCD-induced hepatic dysfunction. SSFP intervention dose-dependently normalized these serum and hepatic parameters, with the most significant reductions observed in the H and T groups relative to the HFHCD control group (*p* < 0.05).

Building upon these findings, we further evaluated the modulatory effects of SSFP on systemic inflammatory status. As shown in Figure 2I–M, serum concentrations of IL-6, IL-18, IL-1β, TNF-α, and MCP-1 were significantly elevated in the HFHCD group relative to the ND group (*p* < 0.05). Similarly, SSFP intervention significantly attenuated systemic low-grade inflammation, as evidenced by dose-dependent suppression of pro-inflammatory cytokines, including IL-6, IL-18, IL-1β, TNF-α, and MCP-1, relative to the HFHCD control group (*p* < 0.05).

### 3.2. SSFP Ameliorates HFHCD-Induced Histopathological Alterations in Multiple Organs

**Figure 3:**
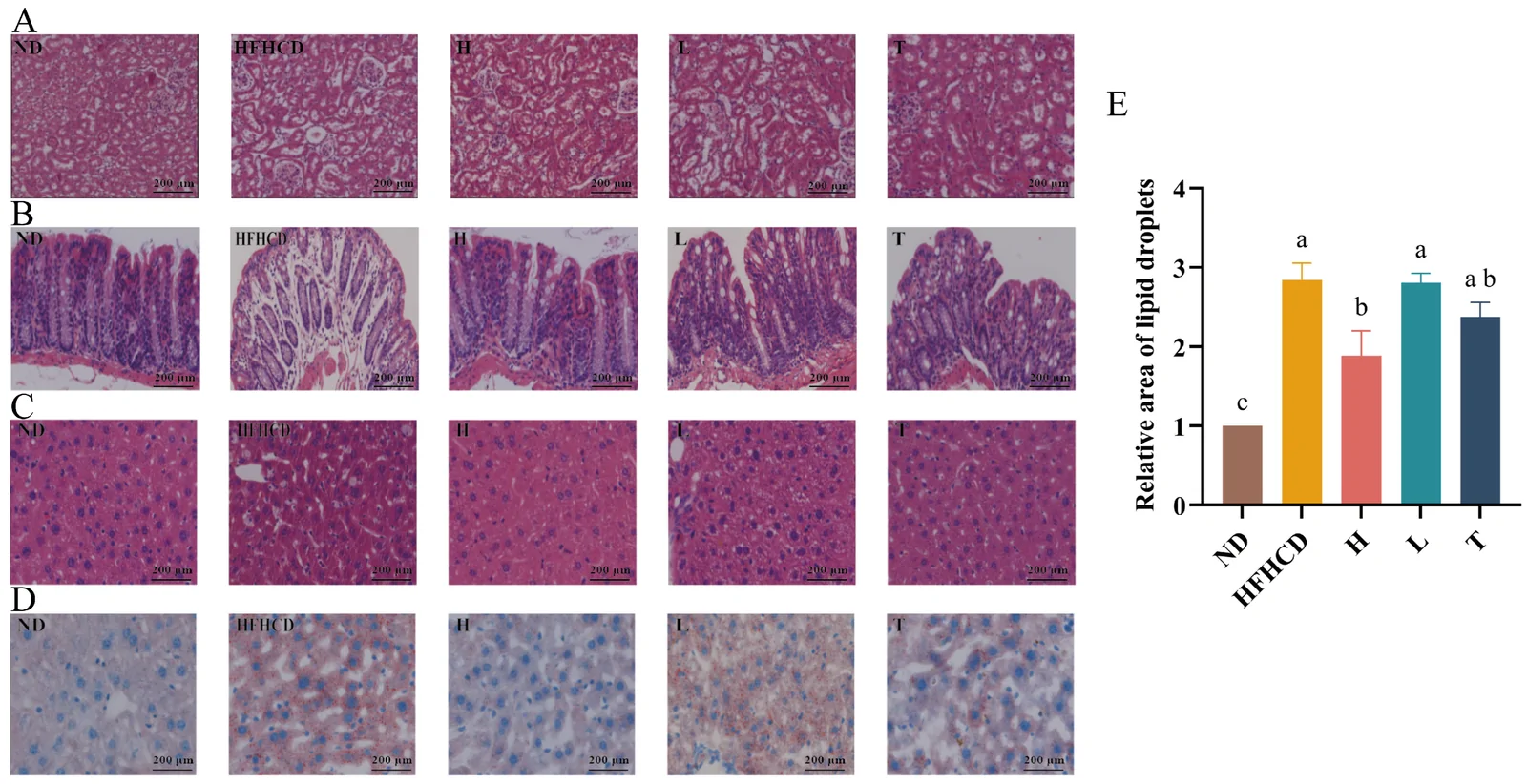
Effects of SSFP on histopathological features and hepatic lipid deposition in HFHCD-fed ApoE^−/−^ mice. (**A**) H&E staining of kidney tissue; (**B**) H&E staining of colon tissue; (**C**) H&E staining of liver tissue; (**D**) Oil Red O staining of liver tissue; (**E**) Quantification of Oil Red O-positive area as a percentage of total hepatic parenchymal area. Scale bar: 200 μm. ND: normal diet group; HFHCD: high-fat, high-cholesterol diet model group; L: HFHCD + SSFP low-dose prevention group (50 mg/kg); H: HFHCD + SSFP high-dose prevention group (100 mg/kg); T: HFHCD + SSFP treatment group (200 mg/kg, intervention starting from the 8th week). Statistical significance versus the HFHCD group was determined by one-way ANOVA with Tukey’s post hoc test: data are means ± SE, n = 3. Different lowercase letters indicate significant differences among groups at the same treatment condition (*p* < 0.05).

To assess the organoprotective effects of SSFP against HFHCD-induced injury, H&E and Oil Red O staining were performed to evaluate histopathological and lipid accumulation changes in liver, kidney, and colon tissues. H&E staining revealed glomerular hypertrophy, vacuolar degeneration of renal tubular epithelial cells, and interstitial inflammatory cell infiltration in the HFHCD group (Figure 3A–C). Colonic mucosal epithelium exhibited disruption, crypt architecture was distorted, goblet cell density was reduced, and inflammatory cell infiltration was evident. Hepatic tissue exhibited diffuse macrovesicular steatosis, characterized by cytoplasmic lipid vacuoles and periportal inflammatory infiltration. Oil Red O staining revealed extensive hepatic lipid accumulation in the HFHCD group relative to the ND group (*p* < 0.05; Figure 3D,E). SSFP intervention dose-dependently ameliorated HFHCD-induced multi-organ histopathological injury, with the H group exhibiting the most pronounced protective effects. Concomitantly, hepatic lipid accumulation and interstitial inflammatory infiltration were significantly attenuated relative to the HFHCD group (*p* < 0.05).

### 3.3. SSFP Modulates the Hepatic GPR120/β-Arrestin-2/NLRP3 Signaling Axis in HFHCD-Fed ApoE^−/−^ Mice

**Figure 4:**
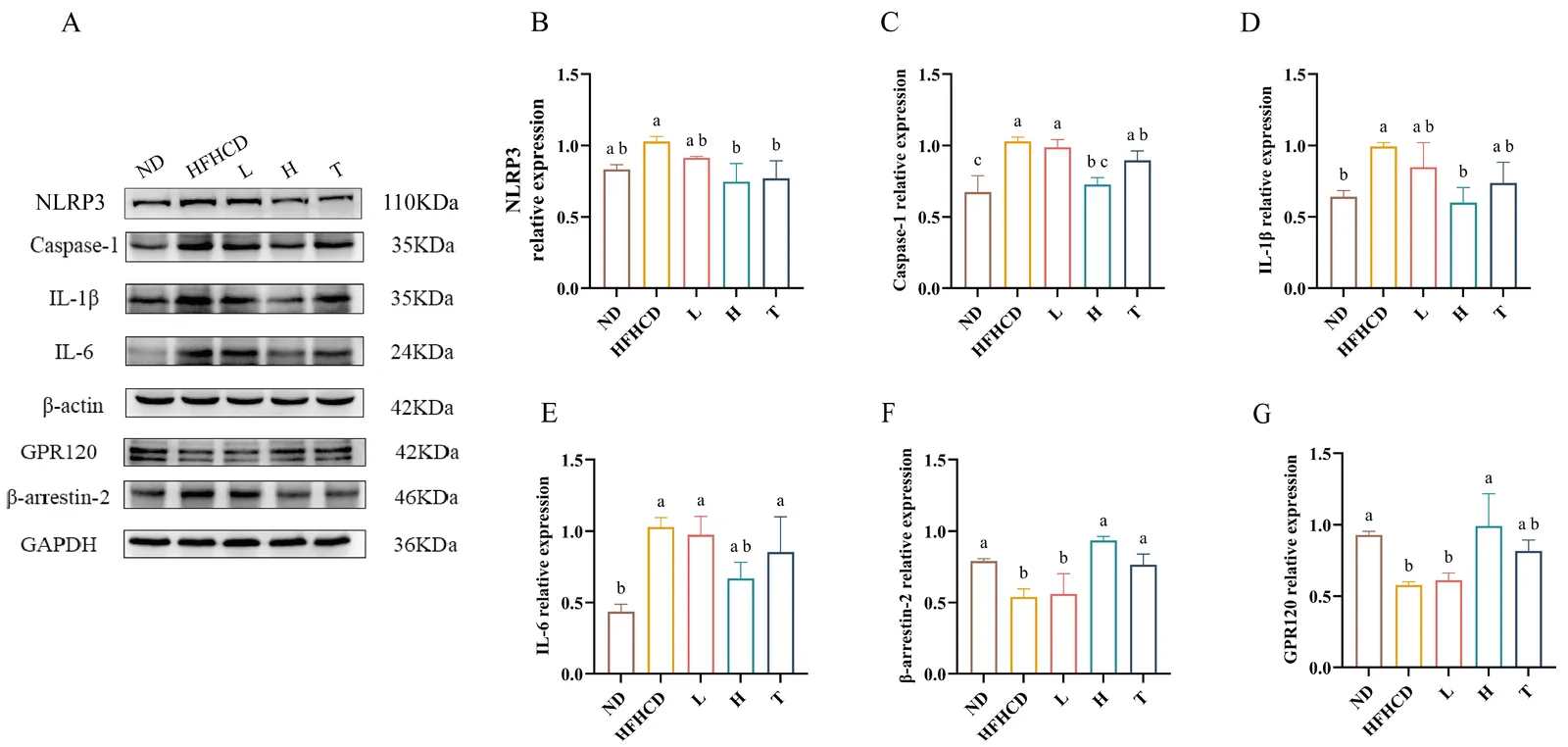
WB analysis of hepatic GPR120, β-arrestin-2, Caspase-1, IL-6, and IL-1β protein expression in HFHCD-fed ApoE^−/−^ mice following SSFP intervention. (**A**) Western blot analysis of various proteins; (**B**) Quantification of NLRP3/β-actin protein ratios; (**C**) Quantification of Caspase-1/β-actin protein ratios; (**D**) Quantification of IL-1β/β-actin protein ratios; (**E**) Quantification of IL-6/β-actin protein ratios; (**F**) Quantification of GPR120/GAPDH protein ratios; (**G**) Quantification of β-arrestin-2/GAPDH protein ratios. ND: normal diet group; HFHCD: high-fat, high-cholesterol diet model group; L: HFHCD + SSFP low-dose prevention group (50 mg/kg); H: HFHCD + SSFP high-dose prevention group (100 mg/kg); T: HFHCD + SSFP treatment group (200 mg/kg, intervention starting from the 8th week). Statistical significance versus the HFHCD group was determined by one-way ANOVA with Tukey’s post hoc test: data are means ± SE, n = 3. Different lowercase letters indicate significant differences among groups at the same treatment condition (*p* < 0.05).

WB was employed to quantify the expression levels of core components of the GPR120 signaling axis—including GPR120, β-arrestin-2, and NLRP3—in hepatic tissue, thereby assessing the involvement of SSFP in modulating inflammation-associated signaling in HFHCD-fed ApoE^−/−^ mice. WB analysis revealed significant downregulation of hepatic GPR120 and β-arrestin-2 protein expression, concomitant with marked upregulation of NLRP3, caspase-1, IL-6, and IL-1β in the HFHCD group relative to the ND group (*p* < 0.05; Figure 4A–G). SSFP intervention dose-dependently enhanced hepatic GPR120 and β-arrestin-2 protein expression, while concurrently suppressing NLRP3, caspase-1, IL-6, and IL-1β levels. And the H and T groups exhibited the most pronounced effects relative to the HFHCD control group (*p* < 0.05; Figure 4A–G). These findings collectively indicate that SSFP attenuates systemic inflammation in HFHCD-fed ApoE^−/−^ mice via modulation of the hepatic GPR120/β-arrestin-2/NLRP3 signaling axis.

Nevertheless, these in vivo findings, based solely on hepatic protein expression, provide correlative evidence supporting the involvement of the GPR120/β-arrestin-2/NLRP3 axis in SSFP-mediated anti-inflammatory effects. However, the upstream molecular triggers initiating this signaling cascade remain to be identified. Building upon prior evidence, the LPC metabolite is hypothesized to function as a potential endogenous ligand for GPR120 within this signaling axis. Subsequently, LPC molecular species exhibiting anti-inflammatory activity will be systematically screened in vitro, and their functional dependence on GPR120 will be validated using selective pharmacological modulators—including the GPR120 agonist compound A and the antagonist AH7614.

### 3.4. LPC 18:1 Functions as a Pivotal Anti-Inflammatory Lipid Mediator Within the Hepatic GPR120 Signaling Axis

**Figure 5:**
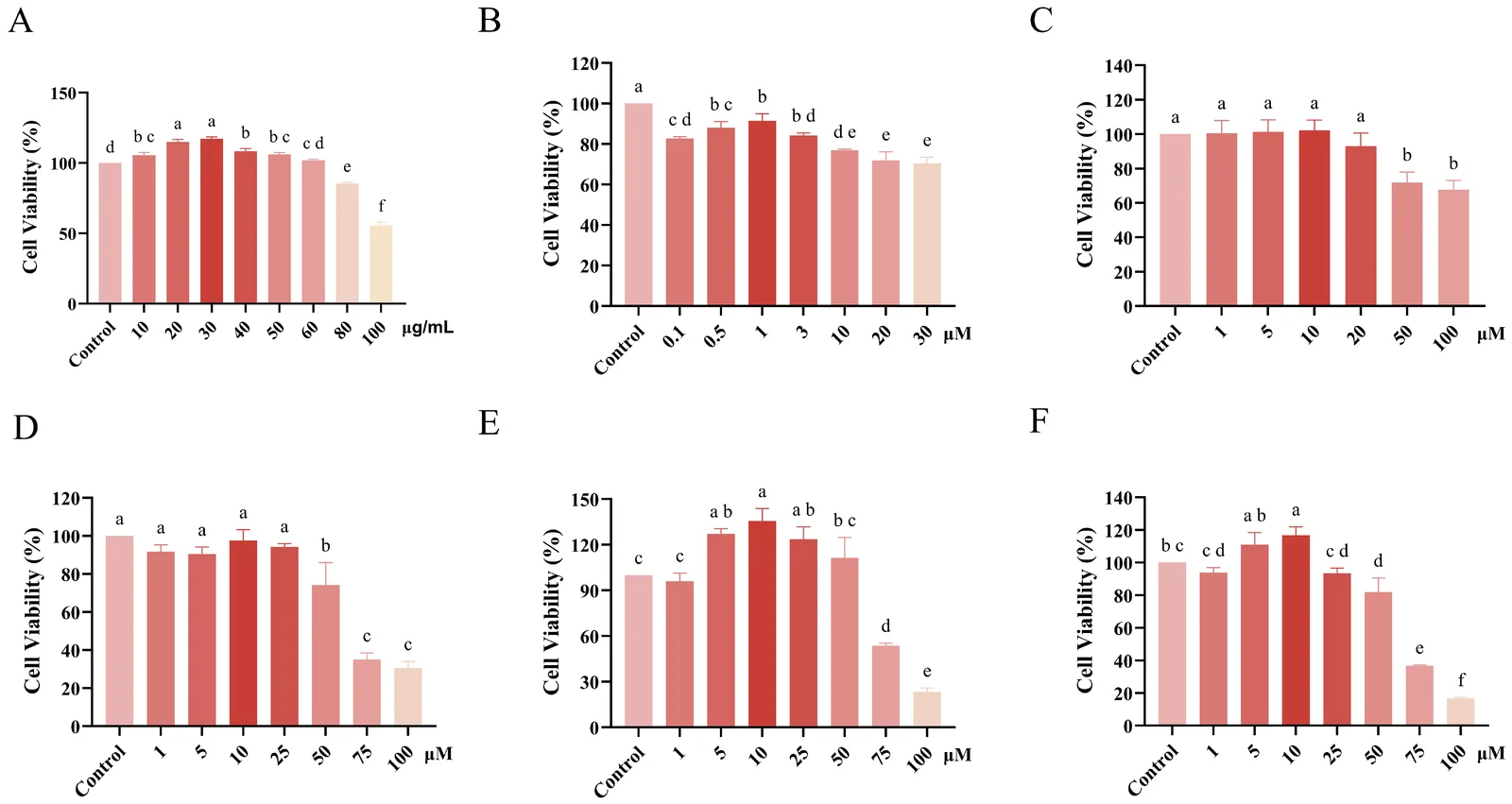
Assessment of RAW264.7 murine macrophage viability based on the CCK-8 assay following exposure to SSFP, TUG-891, AH7614, or individual lysophosphatidylcholine species. (**A**) Dose–response effects of SSFP; (**B**) TUG-891; (**C**) AH7614; (**D**) LPC 14:0; (**E**) LPC 16:0; and (**F**) LPC 18:1. Statistical significance versus the Control group was determined by one-way ANOVA with Tukey’s post hoc test: data are means ± SE, n = 3. Different lowercase letters indicate significant differences among groups at the same treatment condition (*p* < 0.05).

First, cell viability of RAW264.7 murine macrophages was assessed using the CCK-8 assay following exposure to graded concentrations of SSFP, the selective GPR120 agonist TUG-891, the GPR120 antagonist AH7614, and lysophosphatidylcholine species LPC 14:0, LPC 16:0, and LPC 18:1. Based on dose–response viability data, 25 µg/mL and 50 µg/mL were selected as the low- and high-dose concentrations of SSFP, respectively (Figure 5A); 10 μM and 1 μM were adopted as the working concentrations for the GPR120 agonist TUG-891 and antagonist AH7614, respectively (Figure 5B,C); and 10 μM was established as the standardized intervention concentration for all LPC species (LPC 14:0, LPC 16:0, and LPC 18:1) (Figure 5D–F).

**Figure 6:**
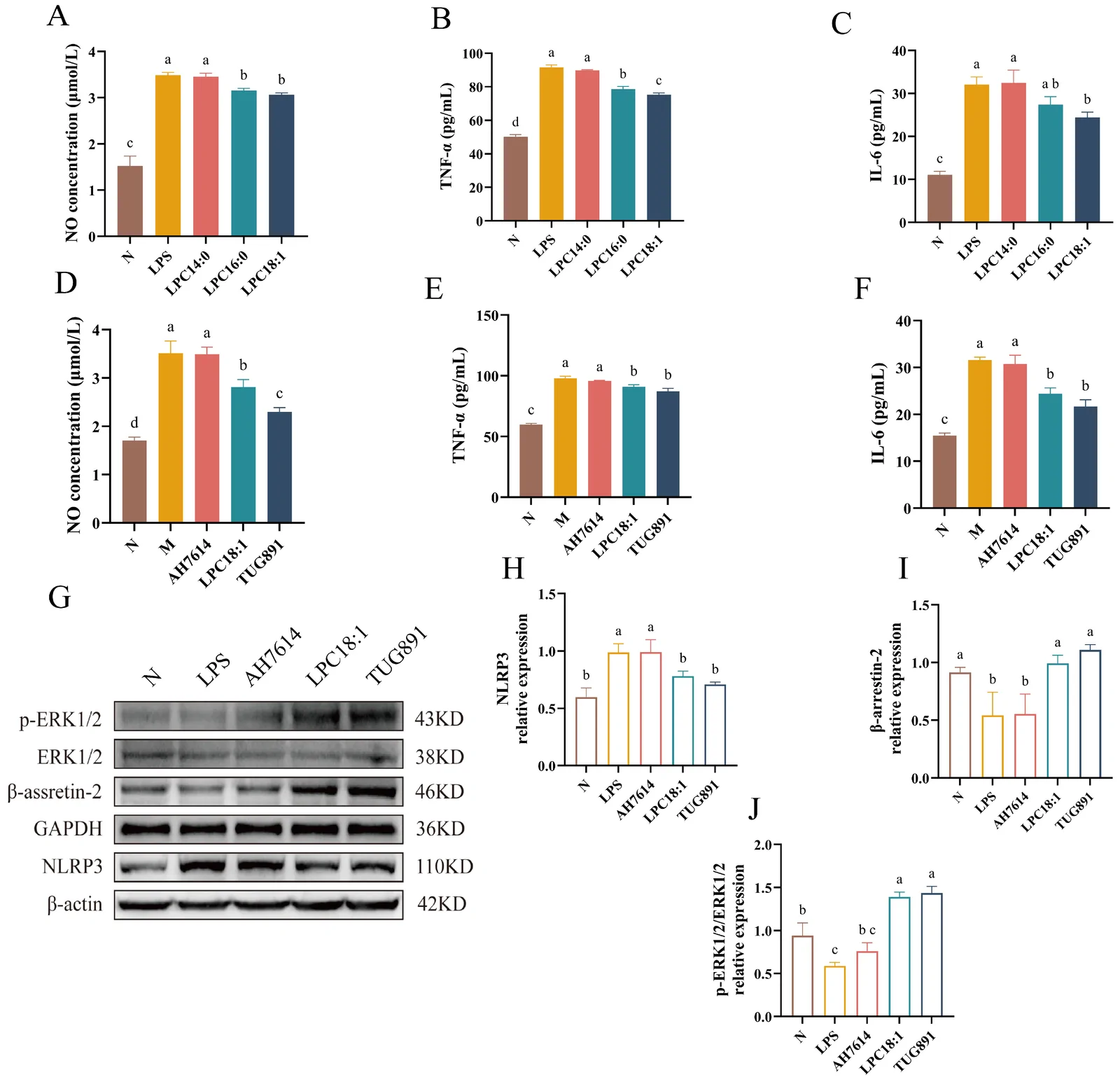
LPC-mediated modulation of inflammatory responses in LPS-stimulated RAW264.7 murine macrophages. (**A**–**C**) Dose-dependent suppression of NO, TNF-α, and IL-6 secretion by distinct LPC molecular species; (**D**–**F**) Potent inhibition of NO, TNF-α, and IL-6 release by LPC 18:1; (**G**–**J**) Western blot analysis and quantitative densitometry of p-ERK1/2, t-ERK1/2, β-arrestin-2, and NLRP3 protein expression in LPC 18:1-treated cells. N: blank control group; LPS: Lipopolysaccharide model group (1 µg/mL); LPC14:0: LPC14:0 intervention group (10 µM); LPC16:0: LPC16:0 intervention group (10 µM); LPC18:1: LPC18:1 intervention group (10 µM); AH7614: GPR120 antagonist group (1 µM AH7614 + 10 µM LPC18:1); TUG891: GPR120 agonist group (10 µM). Statistical significance versus the LPS group was determined by one-way ANOVA with Tukey’s post hoc test: data are means ± SE, n = 3. Different lowercase letters indicate significant differences among groups at the same treatment condition (*p* < 0.05).

Based on previous lipidomics results and literature [20,24], this study further compared the anti-inflammatory activity differences in LPC14:0, LPC16:0, and LPC18:1 in a LPS-induced RAW264.7 cell inflammation model to verify their functional correlation. To mechanistically delineate the upstream lipid mediators underlying SSFP’s anti-inflammatory activity, this study comparatively evaluated the anti-inflammatory efficacy of distinct LPC molecular species, namely LPC 14:0, LPC 16:0, and LPC 18:1, in an LPS-stimulated RAW264.7 murine macrophage model. Relative to the LPS-stimulated control group, LPC 14:0 exerted no statistically significant inhibition of pro-inflammatory cytokine release; in contrast, LPC 16:0 and LPC 18:1 significantly attenuated the LPS-induced secretion of IL-6, IL-1β, and TNF-α (*p* < 0.05; Figure 6A–C). Further comparative analysis revealed that LPC 18:1 exhibited superior efficacy over LPC 16:0 in suppressing LPS-induced NO, IL-6, and TNF-α production. These findings indicate pronounced molecular-species selectivity in the anti-inflammatory activity of LPC, supporting the prioritization of LPC 18:1, as a functionally dominant endogenous GPR120 ligand, for subsequent mechanistic interrogation.

### 3.5. LPC 18:1 Suppresses NLRP3 Inflammasome Activation Through a GPR120/β-Arrestin-2-Dependent Signaling Axis

Having established the potent anti-inflammatory activity of LPC 18:1, this study employed pharmacological modulation, using the selective GPR120 agonist TUG-891 and antagonist AH7614, to determine whether its anti-inflammatory effects are mediated through GPR120-dependent signaling. As shown in Figure 6D–F, LPC 18:1 significantly attenuated LPS-induced NO, IL-6, and TNF-α release (*p* < 0.05 vs. LPS group); notably, the selective GPR120 agonist TUG-891 recapitulated this anti-inflammatory profile with comparable efficacy. In contrast, co-treatment with the GPR120 antagonist AH7614 partially abrogated the suppressive effects of LPC 18:1 on pro-inflammatory cytokine and NO production, confirming that LPC 18:1 exerts its anti-inflammatory activity in a GPR120-dependent manner. WB analysis (Figure 6G–J) revealed that LPS stimulation induced marked dysregulation of inflammatory signaling in RAW264.7 murine macrophages. In contrast, LPC 18:1 treatment significantly enhanced p-ERK1/2 and upregulated β-arrestin-2 protein expression, while concurrently suppressing NLRP3 inflammasome component expression; this regulatory pattern was fully recapitulated by the selective GPR120 agonist TUG-891. Collectively, these findings demonstrate that LPC 18:1 suppresses NLRP3 inflammasome activation, and consequently attenuates the cellular inflammatory response, through GPR120 engagement and subsequent β-arrestin-2–dependent signaling.

### 3.6. SSFP Activates GPR120 Signaling and Attenuates Cellular Inflammatory Responses

**Figure 7:**
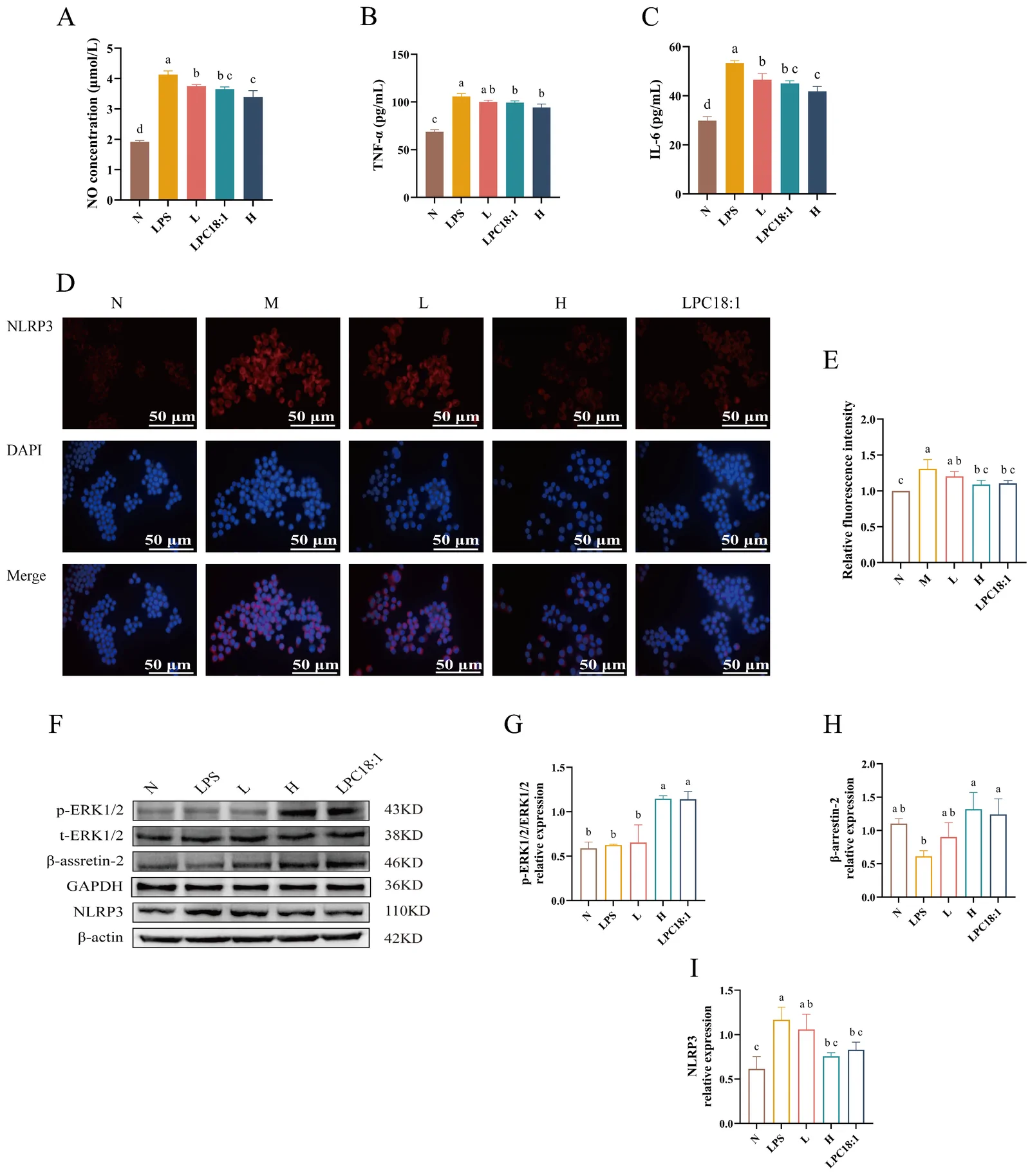
SSFP-mediated modulation of inflammatory responses in LPS-stimulated RAW264.7 murine macrophages. (**A**–**C**) Dose-dependent inhibition of NO, TNF-α, and IL-6 secretion by SSFP; (**D**,**E**) Immunofluorescence localization and quantification of NLRP3 protein expression in SSFP-treated cells (scale bar: 50 μm); (**F**–**I**) WB analysis and quantitative densitometry of p-ERK1/2, t-ERK1/2, β-arrestin-2, and NLRP3 protein levels in SSFP-treated RAW264.7 macrophages. N: blank control group; LPS: Lipopolysaccharide model group (1 µg/mL); LPC18:1: LPC18:1 intervention group (10 µM); SSFP-L: SSFP low-dose intervention group (25 µg/mL); SSFP-H: SSFP high-dose intervention group (50 µg/mL). Statistical significance versus the LPS group was determined by one-way ANOVA with Tukey’s post hoc test: data are means ± SE, n = 3. Different lowercase letters indicate significant differences among groups at the same treatment condition (*p* < 0.05).

To determine whether SSFP recapitulates the GPR120-mediated anti-inflammatory signaling profile of LPC 18:1, this study comparatively evaluated the effects of graded concentrations of SSFP and LPC 18:1 on LPS-induced inflammatory mediator release and on the expression of key GPR120 pathway proteins, including p-ERK1/2, β-arrestin-2, and NLRP3, in RAW264.7 murine macrophages. Quantification of inflammatory mediators revealed that the low- and high-dose SSFP treatment groups and the LPC 18:1 group all significantly attenuated LPS-induced release of NO, IL-6, and TNF-α into the cell supernatant (*p* < 0.05 versus the LPS group; Figure 7A–C). Notably, the H group exhibited superior anti-inflammatory efficacy relative to the LPC 18:1 group in a dose-dependent manner. Immunofluorescence analysis (Figure 7D,E) demonstrated that LPS stimulation significantly enhanced NLRP3 fluorescence intensity in RAW264.7 macrophages relative to the unstimulated control (*p* < 0.05 vs. N group), whereas both the H and LPC 18:1 treatments markedly suppressed NLRP3 signal intensity (*p* < 0.05 vs. LPS group). Notably, the suppressive effect of the H group was significantly more pronounced than that of the LPC 18:1 group. WB analysis (Figure 7F–I) revealed no significant intergroup differences in t-ERK1/2 expression, confirming equal protein loading. In contrast, both the H and LPC 18:1 groups exhibited significant upregulation of p-ERK1/2 and β-arrestin-2 protein levels, alongside marked downregulation of NLRP3 inflammasome component expression, relative to the LPS group (*p* < 0.05). No statistically significant alterations were observed in the L group (*p* > 0.05 vs. LPS group). In conclusion, SSFP activates the GPR120/β-arrestin-2 signaling axis in a dose-dependent manner in RAW264.7 macrophages, leading to suppression of NLRP3 inflammasome assembly and activation. This mechanistic profile is fully concordant with that of LPC 18:1.

## 4. Discussion

Traditionally harvested and processed into a functional herbal tea in local communities, the inflorescences of *Salix songarica* Andersson (*S. songarica*) are renowned for their pharmacological properties, which include heat-clearing, summer-heat-relieving, and gastrointestinal regulatory effects [8]. However, to date, no systematic pharmacological investigation has been conducted to characterize its anti-inflammatory efficacy or elucidate the underlying molecular mechanisms. To the best of our knowledge, this study represents the first systematic evaluation of SSFP’s protective efficacy against systemic inflammation in ApoE^−/−^ mice fed a HFHCD, and provides mechanistic insights into its suppression of NLRP3 inflammasome activation, which is mediated by the GPR120/β-arrestin-2 pathway. In vivo experiments demonstrated that SSFP significantly ameliorated dyslipidemia, hepatic dysfunction, and multi-organ inflammatory injury induced by a HFHCD in ApoE^−/−^ mice, concomitantly enhancing hepatic GPR120/β-arrestin-2 signaling while suppressing NLRP3 inflammasome activation. In vitro functional assays further identified LPC 18:1 as the principal anti-inflammatory bioactive constituent of SSFP, and confirmed that its modulation of the GPR120 signaling axis fully recapitulates the pathway activation profile elicited by SSFP. Collectively, these findings demonstrate that SSFP ameliorates dyslipidemia via multi-target modulation and identify the endogenous lipid metabolite LPC 18:1 as a central mediator of its anti-inflammatory action, thereby establishing a novel mechanistic paradigm for natural polyphenol-based interventions in metabolism-associated chronic inflammation. The above results indicate that SSFP has the potential to serve as a functional food for intervening in human metabolism-related inflammation. However, the bioavailability of polyphenolic components in the human body is a crucial factor determining their actual in vivo effects [36]. *S. songarica* flowers have been consumed locally in Xinjiang as a tea substitute for a long time and have an established preliminary safety foundation for human consumption [7,8]. In the future, it is still necessary to determine its effective dosage range through human pharmacokinetic studies and verify its actual intervention effect in individuals with metabolic inflammation through randomized controlled trials.

This study employed an HFHCD to induce dyslipidemia and chronic low-grade inflammation in ApoE^−/−^ mice, thereby establishing a preclinical model that closely recapitulates the pathophysiological hallmarks of human metabolic disorders [37]. Unlike the LPS-induced acute inflammatory model, HFHCD-triggered inflammation arises from chronic nutrient excess, hepatic cholesterol overload, and systemic accumulation of free fatty acids [38], resulting in elevated serum concentrations of TC, TG, FFA, and LPS. This metabolic endotoxemia promotes Kupffer cell activation, sustained release of pro-inflammatory cytokines, and ectopic lipid deposition in the liver and kidneys [39,40]. SSFP intervention significantly ameliorated serum lipid profiles and hepatic and renal functional parameters, attenuated ectopic lipid accumulation and inflammatory cell infiltration in the liver, kidney, and colon, and collectively indicated that SSFP alleviates HFHCD-induced systemic inflammation via coordinated multi-target regulation of lipid homeostasis, suppression of de novo lipogenesis, and enhancement of intestinal barrier integrity. Recent evidence demonstrates that dietary polyphenols exert synergistic effects in suppressing de novo lipogenesis, enhancing mitochondrial fatty acid oxidation, reinforcing intestinal barrier integrity, and attenuating pro-inflammatory signaling cascades [41,42,43], collectively substantiating the mechanistic plausibility of SSFP’s multi-faceted anti-metainflammatory actions.

Mechanistic investigations have revealed that HFHCD promotes ectopic lipid accumulation and elevates circulating levels of established systemic inflammation biomarkers, such as the key pro-inflammatory cytokines IL-1β, IL-18, and IL-6 [44]. The finding that SSFP intervention normalized serum pro-inflammatory cytokine levels, enhanced hepatic GPR120 and β-arrestin-2 expression, and suppressed NLRP3, caspase-1, and IL-1β expression, suggests that it inhibits NLRP3 inflammasome activation via the GPR120/β-arrestin-2 signaling axis. Among them, IL-1β, as a secreted cytokine downstream of the NLRP3 inflammasome, is susceptible to local inflammatory microenvironment at the tissue level, with a relatively high coefficient of variation, leading to a larger standard deviation in the high-dose group. Nevertheless, its overall downward trend is consistent with the changes in Caspase-1 and IL-6, still supporting the inhibitory effect of SSFP on NLRP3 inflammasome activation. This study observed that after SSFP intervention, the expression of GPR120 and β-arrestin-2 was upregulated in a dose-dependent manner, while the expression of NLRP3, Caspase-1, IL-1β, and IL-6 was significantly downregulated, suggesting a functional association between the GPR120/β-arrestin-2 signaling axis and the inhibition of NLRP3 activation. This synergistic change pattern is consistent with the known negative regulatory mechanism of the GPR120/β-arrestin-2 axis [19,20,21,22]. In vitro functional assays further confirmed the central anti-inflammatory role of LPC 18:1, which exhibited a pharmacological profile consistent with both the synthetic GPR120 agonist TUG-891 and SSFP, specifically by significantly suppressing LPS-induced release of NO, IL-6, and TNF-α, while enhancing p-ERK1/2 and β-arrestin-2 expression and concurrently inhibiting NLRP3 expression. Among these downstream events, ERK1/2 phosphorylation represents a canonical biomarker of GPR120 activation [45], whereas enhanced β-arrestin-2 expression indicates that LPC 18:1 may mediate its anti-inflammatory effects via biased GPR120 signaling. As an endogenous lipid metabolite, LPC 18:1 exerts its anti-inflammatory effects primarily through the GPR120/β-arrestin-2-biased signaling axis, thereby indirectly modulating NLRP3 inflammasome activation. The mechanism of action of LPC 18:1 bears functional resemblance to that of certain natural polyphenols, including curcumin and epicatechin, which ameliorate lipid metabolism disorders through suppression of the NF-κB signaling pathway and downregulation of pro-inflammatory mediators [29]. However, in contrast to the direct NF-κB pathway inhibition mediated by the aforementioned polyphenols, LPC 18:1 modulates NLRP3 inflammasome activation indirectly through GPR120/β-arrestin-2–biased signaling. This mechanistic distinction suggests that natural polyphenols may confer broader anti-inflammatory efficacy in metabolic disease intervention through synergistic multi-target modulation.

Consistent with existing evidence, the flowers of *S. songarica* are a rich natural source of bioactive polyphenols, including epicatechin, luteolin, and gallic acid. Constituents validated in studies of tea polyphenols and other natural polyphenols exert synergistic effects by regulating lipid metabolism [30], enhancing mitochondrial fatty acid oxidation [31], and suppressing pro-inflammatory signaling cascades [46], thereby conferring protection against obesity, non-alcoholic fatty liver disease, and dysregulated glucose and lipid homeostasis. It should be clarified that this study employs SSFP extracts for intervention, aiming to verify the feasibility of the regulatory axis of “polyphenolic compounds—endogenous lipid metabolism remodeling—inflammatory signaling pathway” at the overall level. This discovery reveals a new perspective on the synergistic anti-inflammatory effect of natural polyphenols and endogenous lipid metabolites, providing a theoretical basis for the prevention and treatment of metabolic diseases. However, the specific effects of individual polyphenolic monomers (such as epicatechin and luteolin) in SSFP on the upregulation of LPC18:1 and their direct anti-inflammatory targets remain unclear, which will be a clear direction for subsequent research. However, establishing the overall effectiveness of SSFP and its key signaling axes is a necessary prerequisite for conducting mechanistic studies at the monomer level.

However, this study is subject to several inherent limitations. First, although HFHCD-fed ApoE^−/−^ mice effectively recapitulate the features of metabolic chronic low-grade inflammation, interspecies differences in lipid metabolism and immune regulation between murine models and humans necessitate further clinical validation to establish translational relevance. Second, SSFP constitutes a complex polyphenolic mixture, and the identities of its bioactive monomeric constituents and their synergistic mechanisms remain to be rigorously elucidated through integrated LC-MS/MS–based phytochemical profiling and functional validation assays. Third, this study focused primarily on short-term intervention outcomes; long-term safety profiles, dose–response relationships, and potential off-target effects remain to be systematically characterized. Finally, although LPC 18:1–mediated activation of the GPR120 pathway has been experimentally confirmed, causal validation via genetic ablation (e.g., GPR120 knockout) or targeted knockdown (e.g., RNA interference) remains lacking. Future studies should employ genetically engineered models, such as GPR120^−/−^ mice, to rigorously define the mechanistic centrality of the LPC 18:1/GPR120/β-arrestin-2/NLRP3 axis and to systematically dissect potential synergistic or antagonistic interactions among individual polyphenolic constituents. Such mechanistic insights will inform the rational, precision-based application of natural polyphenols in metabolic disease prevention and intervention.

## 5. Conclusions

This study provides the first systematic characterization of the protective effects of SSFP against HFHCD-induced systemic inflammation in ApoE^−/−^ mice, together with mechanistic insights into its underlying molecular pathways. In vivo experiments demonstrated that SSFP significantly ameliorates dyslipidemia, attenuates multi-organ histopathological injury, and reduces circulating pro-inflammatory cytokine levels. In vitro experiments further confirmed that SSFP suppresses NLRP3 inflammasome activation through upregulation of the key lipid mediator LPC 18:1 and subsequent engagement of the GPR120/β-arrestin-2 signaling axis. This study furnishes mechanistic and translational evidence supporting the functional development of natural polyphenols and advances novel, nutritionally grounded strategies for dietary intervention in metabolic disorders.

## Data Availability

The original contributions presented in this study are included in the article. Further inquiries can be directed to the corresponding authors.
